# Upsurge of dengue outbreaks in several WHO regions: Public awareness, vector control activities, and international collaborations are key to prevent spread

**DOI:** 10.1002/hsr2.2034

**Published:** 2024-04-22

**Authors:** Rapty Sarker, A. S. M. Roknuzzaman, Md. Aminul Haque, Md. Rabiul Islam, Eva Rahman Kabir

**Affiliations:** ^1^ Department of Pharmacy University of Asia Pacific Dhaka Bangladesh; ^2^ School of Pharmacy BRAC University Dhaka Bangladesh

**Keywords:** *Aedes aegypti*, *Aedes albopictus*, climate change, community participation, dengue, dengue virus, pest control

## Abstract

**Background:**

Dengue, the world's fastest‐growing vector‐borne disease, has skyrocketed in the 21st century. Dengue has harmed human health since its first known cases among Spanish soldiers in the Philippines to its 21st‐century outbreaks in Southeast Asia, the Pacific, and the Americas. In light of the current circumstances, it is imperative to investigate its origin and prevalence, enabling the implementation of effective interventions to curb the upsurge.

**Methods:**

Our study examines the history of dengue outbreaks, and evolving impact on public health, aiming to offer valuable insights for a more resilient public health response worldwide. In this comprehensive review, we incorporated data from renowned databases such as PubMed, Google Scholar, and Scopus to provide a thorough analysis of dengue outbreaks.

**Results:**

Recent dengue outbreaks are associated with rapid urbanization, international travel, climatic change, and socioeconomic factors. Rapid urbanization and poor urban design and sanitation have created mosquito breeding places for dengue vectors. Also, international travel and trade have spread the pathogen. Climate change in the past two decades has favored mosquito habitats and outbreaks. Socioeconomic differences have also amplified the impact of dengue outbreaks on vulnerable communities. Dengue mitigation requires vector control, community engagement, healthcare strengthening, and international cooperation.

**Conclusion:**

Climate change adaptation and urban planning are crucial. Although problems remain, a comprehensive vector control and community involvement plan may reduce dengue epidemics and improve public health in our interconnected world.

## 1 BACKGROUND

Dengue fever, a mosquito‐borne viral illness, has left its mark on human history for centuries in tropical regions of Southeast Asia and the Pacific Islands.^1^, ^2^, ^3^, ^4^ Worldwide, dengue epidemics have presented a recurring matter in the public health sector, characterized by intermittent surges in instances and an extension of affected regions. Between 1779 and 1780, Spanish military personnel stationed in the Philippines encountered the initial reported occurrence of a dengue‐like illness. In earlier times, scientists distinguished this ailment by elevated body temperature and intense joint discomfort.^5^, ^6^ The phrase “breakbone fever” originated due to the excruciating pain experienced by afflicted individuals.^7^ During the 19th century, the global expansion of trade and transportation networks significantly facilitated the spread of dengue fever. Therefore, it resulted in epidemics in many port cities across the globe, including regions such as the Caribbean, Central and South America, and Asia.^6^, ^8^ Throughout the 20th century, the global prevalence of dengue continued to increase while the development of effective vaccines remained a challenge. It was not until 1943 that the causal agent of dengue, the dengue virus, was successfully isolated.^9^, ^10^

Nevertheless, efficient transmission and subsequent emergence as a prominent factor contributing to morbidity and mortality primarily occur in tropical and subtropical areas.^3^, ^11^ During the latter part of the 20th century, there was a notable rise in dengue epidemics in Asia and the Americas. Rapid urbanization, population growth, and insufficient public health infrastructure favored an ideal environment for the *Aedes* mosquitoes responsible for transmitting the virus.^12^, ^13^ Dengue cases experienced a notable increase in the 21st century, with significant outbreaks observed in Southeast Asia, the Pacific regions, and the Americas.^3^, ^14^, ^15^ In 2010, the World Health Organization declared dengue a rapidly expanding vector‐borne disease worldwide.^16^ This declaration was based on the significant rise in global incidence, which has increased 30‐fold over the last five decades.^16^, ^17^ Now, dengue is recognized as the most rapidly spreading vector‐borne disease with a substantial impact on global public health. It is evident despite a notable increase of 46% in reported cases between 2015 and 2019.^18^ Brazil, Peru, Mexico, Bangladesh, Bolivia, Nicaragua, Argentina, Colombia, India, and the Philippines have seen the most severe impacts of dengue in 2023 (Table 1).^19^ Among the dengue prevalent countries, Bangladesh has reported the highest number of deaths with the highest case fatality rate (CFR) in 2023.^20^ As of October 2, 2023, over 4.2 million cases and over 3000 fatalities had been documented globally. Autochthonous dengue cases have been reported in Europe, whereas the French Antilles have experienced an epidemic.^19^ Americas region reported a total of 3.1 million dengue cases in 2019, while in 2023, they reported a substantial increase to 3.5 million cases with notable variances across the countries.^19^ It is imperative to prioritize urgent and coordinated preventive measures to address the rising global health crisis. Despite extensive research and ongoing attempts to manage the disease, dengue poses a significant global health obstacle. Further deteriorations may occur due to the lack of a universally accessible vaccine and the advent of severe manifestations of the illness, such as dengue hemorrhagic fever and dengue shock syndrome. The historical account of dengue serves as a poignant reminder of the persistent struggle against infectious hazards in our globally interconnected society.

**Table 1:** Countries with the most dengue cases and deaths during the first 9 months in 2023.

Countries	Confirmed dengue cases	Deaths	Case fatality rate, CFR (%)
Brazil	2,569,746	912	0.04
Peru	264,764	431	0.16
Mexico	216,277	88	0.04
Bangladesh	208,884	1017	0.49
Bolivia	140,246	83	0.06
Nicaragua	134,239	2	0.00
Argentina	123,357	65	0.05
Colombia	94,527	74	0.08
India	94,198	91	0.10
Philippines	80,318	299	0.37

*Source*: ECDC, Dengue worldwide overview. https://www.ecdc.europa.eu/en/dengue-monthly. Data as of October 2, 2023.

In our pursuit of a well‐informed review of dengue outbreaks, we followed a systematic methodology. It commenced with an extensive search across esteemed databases, including PubMed, Google Scholar, and Scopus, to ensure the comprehensiveness and reliability of our sources within the realm of medical and life sciences. Keyword selection was executed thoughtfully, aligning with themes prevalent in the existing literature on dengue outbreaks, incorporating MeSH database‐derived keywords, which include, dengue virus, dengue outbreaks, vector control, and climate change. Our research encompassed articles from diverse timeframes, stretching from the earliest accounts of dengue's history to the latest studies up to 2023, primarily emphasizing articles published between 2016 and 2023. Our data collection extended beyond peer‐reviewed journals, incorporating reputable sources and authentic websites to enhance the breadth and reliability of our review. Our primary focus lay on comprehensively dissecting the historical evolution of dengue outbreaks and their implications for public health, with the ultimate aim of providing valuable insights to bolster disaster risk assessment, emergency health management, and global public health response, fostering increased resilience in the face of this public health challenge.

## 2 POSSIBLE REASONS BEHIND THE FREQUENT DENGUE OUTBREAKS

The frequency of dengue epidemics has increased globally in recent years, primarily due to various causes that intersect, including environmental, demographic, and climate‐related shifts.^21^, ^22^ Urbanization has emerged as a significant driving force, given the ongoing global migration of the population toward urban centers. This phenomenon has led the establishment of densely populated regions that offer favorable conditions for the proliferation of *Aedes* mosquitoes, known as dengue virus vectors.^23^, ^24^ Insufficient urban planning, inadequate water storage techniques, and substandard sanitation systems in numerous metropolitan areas have contributed to the proliferation of mosquito breeding places, hence generating optimal circumstances for the spread of dengue. Furthermore, there has been a significant increase in international travel and trade, which has resulted in infected individuals transporting the virus across national boundaries. It has led to the emergence of novel viral strains that dramatically facilitated the swift dissemination of the disease.^25^ The adaptability of the dengue virus, characterized by the presence of four unique serotypes, poses significant challenges to control.^26^, ^27^ This is because patients who have previously recovered from one serotype remain vulnerable to infection by the other serotypes, which may result in more severe manifestations of the disease during subsequent outbreaks.^28^, ^29^

Furthermore, the low or unavailability of healthcare support in numerous places hampers timely identification and treatment that exacerbates the impact of dengue outbreaks.^30^, ^31^ The lack of a universally accessible and efficacious dengue vaccine, along with public resistance or reticence toward immunization initiatives, has rendered numerous susceptible communities without protection.^32^ Climate factors have a significant impact on the transmission of dengue. The life cycle and behavior of *Aedes* mosquitoes, which serve as vectors for transmitting the virus, are influenced by temperature and rainfall patterns. Elevated temperatures expedite the maturation of mosquitoes and reduce the duration of viral incubation within them. The upsurge with heightened precipitation results in breeding habitats, such as stagnant water contained within receptacles, serve as suitable locations for mosquitoes to deposit their eggs.^33^, ^34^

On the contrary, prolonged periods of aridity followed by substantial precipitation can elicit abrupt escalations in mosquito populations.^35^, ^36^ Climate change contributes to the intensification of these processes, enlarging the geographical distribution of *Aedes* mosquitoes, and modifying the temporal patterns of dengue outbreaks.^36^, ^37^ The exponential expansion of urban centers and metropolitan regions has fostered an environment conducive to transmitting dengue fever. Dense populations facilitate the persistent availability of human hosts for mosquitoes, hence augmenting the probability of virus transmission.^38^, ^39^ Furthermore, unplanned urbanization results in insufficient sanitation, waste management, and water storage methods, generating many breeding grounds for *Aedes* mosquitoes within residential areas.^40^, ^41^ Inadequately governed urban settings can potentially exacerbate the magnitude of dengue fever epidemics. Household water storage methods play a crucial role in dengue outbreaks.^40^, ^41^ As breeding habitats, *Aedes* mosquitoes utilize various receptacles, such as buckets, jars, and abandoned tires.^42^ These mosquitoes prefer depositing their eggs in small, dimly lit receptacles containing stagnant water. The absence of a dependable piped water supply in certain regions amplifies the issue due to the increased need for water storage. Implementing public awareness campaigns that advocate for the appropriate storage of water and the frequent cleaning of containers is of utmost importance in mitigating the proliferation of mosquito breeding sites.

The pathophysiology of the recent dengue outbreak has demonstrated a significant change, leading to a modified fatality profile compared to past outbreaks.^43^, ^44^ The dynamic nature of the pathophysiology of dengue has prompted a reassessment of our comprehension of the illness and a readjustment of public health interventions.^45^ During this outbreak, a notable alteration in the pathophysiology of dengue is the occurrence of unconventional clinical manifestations. Although classical signs of dengue, such as elevated body temperature, intense joint discomfort, and skin rash, continue to be commonly observed, there has been a rise in the occurrence of atypical presentations.^46^, ^47^, ^48^, ^49^ These encompass gastrointestinal manifestations, neurological sequelae, and cardiac implications, showing a relatively low incidence in prior epidemic occurrences. The broadened range of clinical manifestations associated with dengue fever has presented difficulties in diagnosing the disease, highlighting the importance of continuous monitoring and revised clinical protocols.^50^

Moreover, the intensity of dengue cases observed throughout this epidemic has demonstrated a nuanced trend.^45^, ^51^ While the overall CFR has not experienced a significant increase, there has been a notable alteration in the demographic composition of severe cases.^19^, ^52^ In the past, the incidence of severe dengue was predominantly observed in the pediatric and young adult populations.^53^ However, it has been found that a higher proportion of severe cases in this outbreak are occurring among older people who have comorbidities.^54^, ^55^ The information has inspired a reassessment of risk elements and emphasizes the significance of customized therapeutic administration for distinct age cohorts.

Furthermore, the fatality rate has been significantly higher for individuals experiencing subsequent dengue infections in the current outbreak.^56^, ^57^ Individuals previously infected with dengue are more susceptible to acquiring severe manifestations of the disease, resulting in a greater fatality rate among this population. This observation underscores the intricate interaction between the immune response of the host and the many serotypes of the dengue virus, underscoring the significance of immunity particular to each serotype in developing dengue‐related physiological abnormalities. We observed the impact of healthcare system capacity and resources on the mortality profile of this dengue outbreak. The strain on healthcare systems has postponed the medical services for specific individuals, notably those residing in heavily populated regions.^55^ The increased mortality rates observed among persons with severe dengue can be attributed to delayed access to healthcare, highlighting the significant impact of healthcare infrastructure on mitigating dengue‐related fatalities.^56^, ^57^

The current dengue outbreak has demonstrated notable alterations in its pathophysiology, characterized by unusual clinical manifestations, a change in the distribution of severe cases across different age groups, and a heightened influence of secondary infections. The changes in the pathophysiological characteristics of dengue and the difficulties faced by healthcare systems have impacted the patterns of mortality associated with this disease. As a result, it is necessary to develop a comprehensive and flexible public health strategy that can effectively respond to the changing dynamics of dengue.

## 3 PUBLIC HEALTH CHALLENGES DUE TO DENGUE OUTBREAKS

Dengue epidemics pose complex public health challenges that have extensive ramifications. One notable aspect of these challenges pertains to the burden they impose on the infrastructure of healthcare. During epidemics, there is frequently a shortage of resources due to the increase in hospitalizations and demand for medical care.^58^ This shortage impacts not only the treatment of dengue patients but also individuals with unrelated medical conditions. Furthermore, it is crucial to acknowledge the significant financial consequences that epidemics of dengue can cause. Affecting regions and communities severely, the considerable costs associated with increased healthcare expenditures, productivity losses, and decreased economic activity as a result of illness and reduced work hours can impede economic growth.^58^, ^59^, ^60^

The growing number of severe dengue complications, including dengue shock syndrome and dengue hemorrhagic fever, further complicate the public health dilemma. These severe manifestations of the illness require extensive medical intervention and frequently lead to increased mortality rates, especially among populations that are more susceptible.^61^ In addition, the worldwide dissemination of dengue is a substantial issue, given that it not only complicates the management of local outbreaks but also promotes transmission across borders, thereby posing a threat to public health on a global scale. The capacity of dengue to surpass geographical limitations adds an additional layer of complexity to control endeavors.^31^

Socioeconomic inequalities significantly impact dengue, which disproportionately impacts low and middle‐income nations.^60^, ^62^ This strain places additional strain on healthcare systems that are already precarious, further exacerbating preexisting health disparities. Marginalized communities that have restricted healthcare access and preventive measures bear a greater burden from the disease. Dengue outbreaks not only have adverse effects on individual health but also present significant obstacles to daily life, impeding educational and occupational prospects, as well as impeding economic expansion and social progress in regions where they are endemic.^63^ Moreover, even in individuals who recover from the acute phase of the illness, dengue can cause enduring health complications, such as post‐dengue fatigue syndrome and other persistent health problems. In areas heavily dependent on tourism, dengue epidemics may result in a reduction in the volume of visitors, which may have adverse effects on the local economy and potentially compromise the provision of healthcare services.

## 4 MITIGATION STRATEGIES AGAINST DENGUE UPSURGES ACROSS THE COUNTRIES

**Figure 1:**
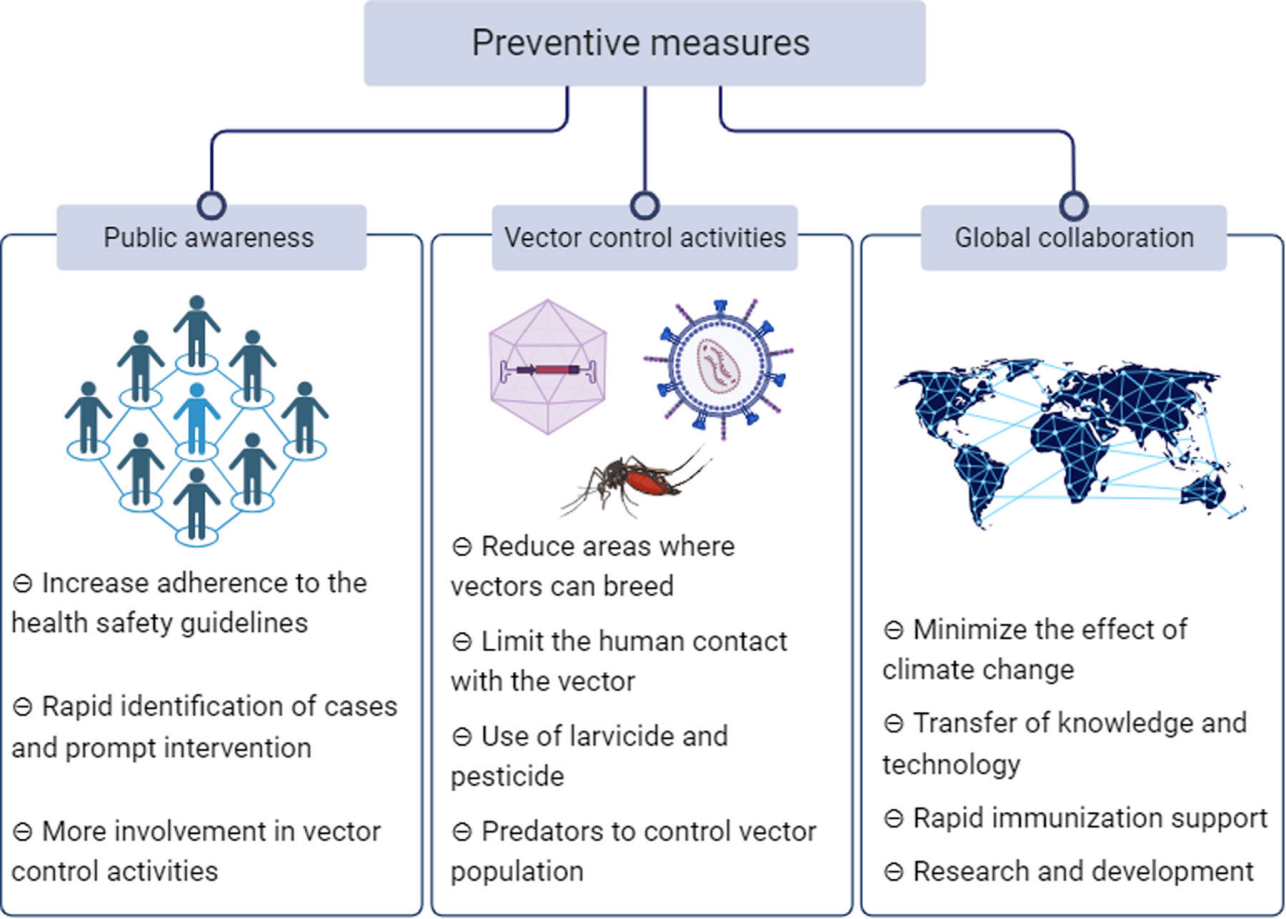
Key preventive measures against dengue outbreaks across the countries.

Addressing dengue outbreaks presents a varied and intricate task that necessitates implementing a comprehensive strategy encompassing public health measures, community engagement, and international collaboration (Figure 1). Governments can take several crucial initiatives to address the dengue issue. The implementation of efficient vector control measures for the mitigation of dengue.^64^, ^65^ This encompasses the administration of *Aedes* mosquito populations using strategies such as the application of insecticides, the decrease of larval sources, and the utilization of mosquito nets and screens.^66^, ^67^ The active participation of the community is of utmost importance in identifying and eradicating breeding sites located within residential areas, educational institutions, and public areas.^68^, ^69^, ^70^ The implementation of Integrated Pest Management (IPM) strategies necessitates a comprehensive approach to control mosquitoes. It encompasses environmental improvements, such as enhanced urban planning strategies to mitigate water accumulation and use biological control approaches utilizing natural predators of *Aedes* mosquitoes. IPM solutions employ techniques that minimize reliance on chemical pesticides, mitigating the risk of resistance development.^71^, ^72^ It is imperative to emphasize enhancing public understanding regarding dengue transmission, prevention, and control.^73^ Educational campaigns can disseminate knowledge within communities regarding the potential hazards associated with dengue, techniques for identifying sites conducive to mosquito breeding, and the need to adopt personal protective measures, such as donning long‐sleeved attire and employing mosquito repellents.

The rapid identification of dengue cases is of utmost importance for prompt intervention.^74^ It is recommended that nations allocate resources to establish comprehensive surveillance systems to effectively monitor the prevalence of dengue, analyze disease patterns, and identify areas of concentrated transmission. The widespread availability of diagnostic tests that are both accessible and accurate is essential in promptly confirming cases and distinguishing between dengue and other febrile diseases.^65^, ^74^, ^75^ It is imperative to enhance healthcare systems to manage dengue cases successfully.^76^ This includes education and training for healthcare professionals to enhance their ability to identify and effectively treat severe cases of dengue fever. Additionally, it involves the establishment of a sufficient inventory of intravenous fluids and the enhancement of healthcare service accessibility, particularly in regions that are susceptible and geographically isolated. Sustained investigation into the field of dengue, encompassing the advancement of vaccines, strategies for vector management, and the development of innovative diagnostic techniques, is imperative for effectively mitigating the impact of this disease in the long run.^77^, ^78^ Promising advancements for dengue prevention involve genetically engineered insects that effectively diminish mosquito populations.

The global spread of dengue fever disregards geographical boundaries, emphasizing the indispensable nature of international cooperation. Nations must exchange information and experiences about the prevention and management of dengue. Regional organizations and global health authorities can enhance technical support.^79^ The epidemic preparation plans are of utmost importance to facilitate a prompt and efficient response to dengue outbreaks. The proposed plans should encompass comprehensive protocols for case management, vector control, and communication techniques. Considering the significant impact of climate on the spread of dengue,^21^, ^80^ it is imperative for nations to include climate change adaptation methods in their endeavors to mitigate dengue. Surveillance of climate patterns, predicting disease outbreaks, and adjusting vector control strategies in response to evolving environmental circumstances.

As previously stated, the implementation of vector control measures is of utmost importance in the attempts to mitigate dengue. Therefore, nations employ various tactics to combat the *Aedes* mosquitoes. Indoor residual spraying of insecticides is a widely used method for mitigating adult mosquito populations by specifically targeting resting and flying mosquitoes. Nevertheless, overutilization of insecticides can result in the development of mosquito resistance and give rise to environmental apprehensions.^81^ Larval source reduction entails the eradication of breeding grounds for mosquitoes by promoting the identification and elimination of stagnant water containers within communities, which serve as deposition sites for *Aedes* mosquitoes' eggs.^82^ This grassroots initiative enables individuals to assume accountability for their immediate surroundings. Biological control strategies involve the introduction of indigenous predators of *Aedes* mosquitoes, such as specific fish species or copepods, into aquatic environments, hence reducing reliance on chemical insecticides.^83^ Promising strategies in mitigating mosquito populations involve implementing innovative methods such as releasing genetically modified mosquitoes.^84^ These genetically modified organisms possess genes that diminish the survivability and capacity of the virus to transmit.^85^ Community‐based interventions are of utmost importance as they involve local populations in vector control. Educational and training programs can provide the necessary knowledge and skills to the general people to detect and eradicate breeding places. Additionally, these interventions also emphasize the adoption of personal protective measures such as the use of mosquito nets and screens. These initiatives promote a feeling of responsibility in the context of dengue prevention.

Community involvement is of equal importance in the efforts to combat dengue. It enables individuals and communities to engage actively in the prevention of dengue. Public awareness campaigns employ a range of media platforms, such as radio, television, posters, and social media, to provide information to communities regarding the transmission and preventive measures of dengue, assuring a wide‐ranging impact.^86^ The organization of clean‐up efforts in dengue‐prone areas, aimed at eliminating potential breeding sites and fostering collective action, is significantly influenced by community mobilization led by local leaders, schools, and community organizations. Behavioral modification initiatives prioritize altering behaviors that contribute to the spread of dengue fever. These efforts aim to promote the adoption of preventive measures.^87^ The ultimate objective is to minimize individuals' susceptibility to mosquito bites. Engaged communities serve as proactive mechanisms for early detection and reporting of potential dengue cases and mosquito breeding sites. This active involvement significantly assists public health authorities in effectively directing their efforts toward implementing targeted vector control measures. Community health workers who have received training play a crucial role in connecting communities with healthcare systems. They fulfill this role by imparting knowledge to locals on preventing dengue, disseminating educational materials, and easing access to healthcare services.^76^

## 5 CONCLUSION

Dengue history reflects the enduring battle against infectious diseases in our interconnected world. Emerging in 18th‐century Southeast Asia, dengue has evolved into a global health concern with recurrent outbreaks, especially in the 21st century. Urbanization, international travel, climate change, and socioeconomic factors have fueled recent dengue upsurges. Rapid urban growth has created mosquito‐friendly environments, and global travel has facilitated virus spread. Climate change has expanded mosquito habitats. Socioeconomic disparities amplify dengue's impact. Countries need multifaceted strategies, including robust vector control, community engagement, healthcare strengthening, and international collaboration to mitigate recent dengue upsurges. Adapting to climate change and addressing urban planning are crucial. Despite challenges, a comprehensive approach combining vector control with community involvement holds promise for fewer outbreaks and improved public health in our interconnected world.

## AUTHOR CONTRIBUTIONS

**Rapty Sarker**: Data curation; writing—original draft. **A. S. M. Roknuzzaman**: Writing—original draft; data curation. **Md. Aminul Haque**: Validation; writing—review and editing. **Md. Rabiul Islam**: Conceptualization; supervision; writing—review and editing. **Eva Rahman Kabir**: Conceptualization; supervision; writing—review and editing.

## CONFLICT OF INTEREST STATEMENT

The authors declare no conflict of interest.

## TRANSPARENCY STATEMENT

The lead author Md. Rabiul Islam affirms that this manuscript is an honest, accurate, and transparent account of the study being reported; that no important aspects of the study have been omitted; and that any discrepancies from the study as planned (and, if relevant, registered) have been explained.

## Data Availability

Data sharing is not applicable as no new data is generated.
